# Phylogenetic analysis of SARS-CoV-2 in Boston highlights the impact of superspreading events

**DOI:** 10.1126/science.abe3261

**Published:** 2020-12-10

**Authors:** Jacob E. Lemieux, Katherine J. Siddle, Bennett M. Shaw, Christine Loreth, Stephen F. Schaffner, Adrianne Gladden-Young, Gordon Adams, Timelia Fink, Christopher H. Tomkins-Tinch, Lydia A. Krasilnikova, Katherine C. DeRuff, Melissa Rudy, Matthew R. Bauer, Kim A. Lagerborg, Erica Normandin, Sinéad B. Chapman, Steven K. Reilly, Melis N. Anahtar, Aaron E. Lin, Amber Carter, Cameron Myhrvold, Molly E. Kemball, Sushma Chaluvadi, Caroline Cusick, Katelyn Flowers, Anna Neumann, Felecia Cerrato, Maha Farhat, Damien Slater, Jason B. Harris, John A. Branda, David Hooper, Jessie M. Gaeta, Travis P. Baggett, James O’Connell, Andreas Gnirke, Tami D. Lieberman, Anthony Philippakis, Meagan Burns, Catherine M. Brown, Jeremy Luban, Edward T. Ryan, Sarah E. Turbett, Regina C. LaRocque, William P. Hanage, Glen R. Gallagher, Lawrence C. Madoff, Sandra Smole, Virginia M. Pierce, Eric Rosenberg, Pardis C. Sabeti, Daniel J. Park, Bronwyn L. MacInnis

**Affiliations:** 1 Broad Institute of Harvard and MIT, 415 Main Street, Cambridge, MA 02142, USA.; 2 Division of Infectious Diseases, Massachusetts General Hospital, Boston, MA, USA.; 3 Department of Organismic and Evolutionary Biology, Harvard University, Cambridge, MA 02138, USA.; 4 Department of Immunology and Infectious Diseases, Harvard T.H. Chan School of Public Health, Harvard University, Boston, MA, USA.; 5 Massachusetts Department of Public Health, Boston, MA, USA.; 6 Harvard Program in Biological and Biomedical Sciences, Harvard Medical School, Boston, MA 02115, USA.; 7 Department of Systems Biology, Harvard Medical School, Boston, MA, USA.; 8 Department of Pathology, Massachusetts General Hospital, Boston, MA, USA.; 9 Department of Biomedical Informatics, Harvard Medical School, Boston, MA, USA.; 10 Division of Pulmonary and Critical Care, Massachusetts General Hospital, Boston, MA, USA.; 11 Department of Pediatrics, Harvard Medical School, Boston, MA, USA.; 12 Institute for Research, Quality, and Policy in Homeless Health Care, Boston Health Care for the Homeless Program, Boston, MA, USA.; 13 Section of General Internal Medicine, Boston University Medical Center, Boston.; 14 Division of General Internal Medicine, Massachusetts General Hospital, Boston.; 15 Department of Medicine, Harvard Medical School, Boston, MA, USA.; 16 Institute for Medical Engineering and Sciences, Massachusetts Institute of Technology, Cambridge, MA 02139, USA.; 17 Program in Molecular Medicine, University of Massachusetts Medical School, Worcester, MA 01605, USA.; 18 Massachusetts Consortium on Pathogen Readiness, Boston, MA, 02115, USA.; 19 Center for Communicable Disease Dynamics, Department of Epidemiology, Harvard T. H. Chan School of Public Health, Boston, MA 02115, USA.; 20 University of Massachusetts Medical School, Infectious Diseases and Immunology, Worcester, MA 01655.; 21 Pediatric Infectious Disease Unit, Massachusetts General Hospital for Children, Boston, MA, USA.; 22 Department of Pathology, Harvard Medical School, Boston, MA, USA.; 23 Howard Hughes Medical Institute, 4000 Jones Bridge Rd, Chevy Chase, MD 20815.

## Abstract

Analysis of 772 complete SARS-CoV-2 genomes from early in the Boston area epidemic revealed numerous introductions of the virus, a small number of which led to most cases. The data revealed two superspreading events. One, in a skilled nursing facility, led to rapid transmission and significant mortality in this vulnerable population but little broader spread, while other introductions into the facility had little effect. The second, at an international business conference, produced sustained community transmission and was exported, resulting in extensive regional, national, and international spread. The two events also differed significantly in the genetic variation they generated, suggesting varying transmission dynamics in superspreading events. Our results show how genomic epidemiology can help understand the link between individual clusters and wider community spread.

SARS-CoV-2 has now caused over fifty million infections and over one million reported deaths (*1*) in one of the worst public health crises of the past century. Cases are currently surging to unprecedented levels in the United States, reaching over 180,000 cases reported daily during November 2020. Massive ongoing transmission globally underscores that most countries have not found effective ways to control spread of the virus; better understanding of transmission dynamics could contribute to more targeted and effective responses to the pandemic. Reports of COVID-19 transmission have featured clusters of cases linked to gatherings, including ones in workplaces (*2*) and churches (*3*), and especially in close living environments such as care homes (*4*) and homeless shelters (*5*). These clusters are thought to often involve superspreading (*6*, *7*), in which one individual infects many others (defined here as more than eight secondary cases; see Materials and Methods), yet the contribution of these events to regional and national transmission is not well understood. Instead, the evidence indicating that case clusters and superspreading events are major drivers of transmission has been based largely on time-series data showing an increase in cases following them (*8*), which has limited ability to determine the contribution of any event to overall transmission. Contact tracing from such events can be similarly uninformative, as it is resource intensive, invasive, and often limited in scope. Likewise, without genetic data about the viruses involved, it is often not possible to distinguish superspreading events from other forms of locally intense transmission, or from cases that occur in close proximity by chance. Yet understanding the role of superspreading events in transmission is critical for prioritizing public health interventions. To further that understanding, we used genomic epidemiology to investigate the introduction and spread of SARS-CoV-2 in the Boston, Massachusetts (MA) area, which was severely affected in the first wave of the pandemic. These data allowed us to study early outbreak dynamics and to examine the role of importations and superspreading events in fueling epidemic spread.

## Genomic epidemiology of Boston superspreading events

**Fig. 1:**
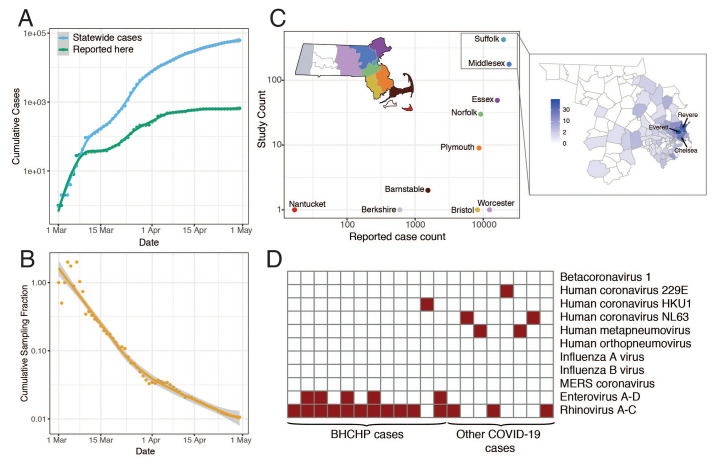
Epidemiology of SARS-CoV-2 in Massachusetts and of sequenced viral genomes. (**A**) Cumulative confirmed and presumed cases reported state-wide in MA (*10*) from March 1 through May 1, 2020, and the number of these cases that successfully yielded complete genomes with >98% coverage (green) in this study. (**B**) Cumulative proportion of all MA confirmed positive cases with complete genome sequences from unique individuals that are part of this dataset over time. (**C**) Total number of cases compared to cases in this study by MA county. Points are colored by state as shown in the state map. Suffolk and Middlesex counties are shown in detail to the right with counts from this study shown by zip code. (**D**) Detection of common respiratory viruses from metagenomic sequencing data. Samples with >10 reads mapped to at least 1 of these viruses using Kraken2 are shown in red. Enterovirus and Rhinovirus species have been grouped owing to the difficulty in discriminating at the sequence level.

The first known case in the Boston area was confirmed on February 1, 2020 (*9*); case counts rapidly increased through March and peaked in the third week in April. We performed viral genome sequencing and phylogenetic analysis of SARS-CoV-2-positive nasopharyngeal (NP) samples collected between March 4th and May 9th, 2020 by the Massachusetts Department of Public Health (MADPH) and Massachusetts General Hospital (MGH). Our dataset includes nearly all confirmed early cases of the epidemic (Fig. 1, A and B); samples from many of the highest-prevalence communities in the Boston area across the first wave (Fig. 1C), including Chelsea, Revere, and Everett (Fig. 1C and fig. S1); and samples from putative superspreading events involving an international conference and congregate living environments, specifically among residents and staff at a skilled nursing facility and in homeless shelters. As seen elsewhere, close-quarters living facilities like these have been disproportionately affected by COVID-19 in MA, accounting for 22% of confirmed cases and 64% of reported deaths through August 1, 2020 (*10*).

We generated 778 complete SARS-CoV-2 assemblies (>98% complete) from 772 individuals, and an additional 72 partial genomes (>80% complete), using Illumina-based unbiased metagenomic short-read sequencing, followed by reference-guided assembly using viral-ngs 2.0.21 software (*11*) with the Wuhan-Hu-1 sequence (NC_045512.2) as the reference (Materials and Methods). Genome recovery and coverage were strongly correlated with viral abundance (fig. S2) and clinical diagnostic test results (fig. S3). Genomes were separated from one another by a median of 6 single nucleotide polymorphisms (SNPs) (interquartile range 4-9 SNPs; range 0-85 SNPs) (fig. S4, A and B). As expected during rapid population expansion, most alleles were rare, as assessed by a strongly negative Tajima’s D statistic throughout the genome (fig. S4C). In 20 samples (1.4% of sequenced cases) we identified the presence of at least one other common respiratory pathogen (Fig. 1D) via sequencing and confirmed it with a second assay (fig. S5). Co-infections were more commonly detected in residents and staff of homeless shelters (12/314) than in the other cases in the study (8/1117) (*P* = 0.0002, Fisher’s exact test).

**Fig. 2:**
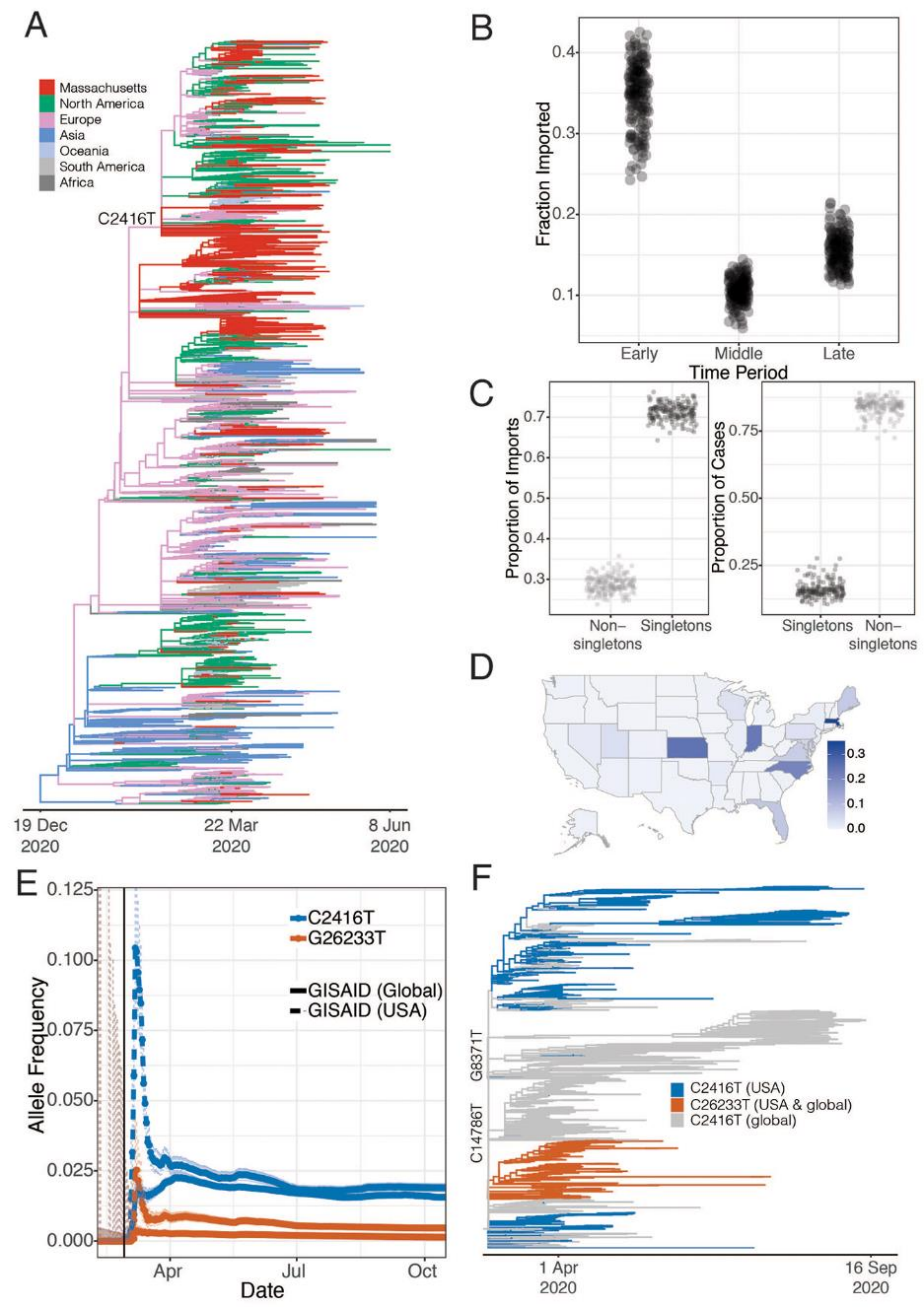
Introductions of SARS-CoV-2 into Massachusetts. (**A**) Time tree of 772 MA genomes and a global set of 4,011 high-quality genomes from GISAID. To view an interactive version of this tree and for more information on specific sub-groupings within the MA dataset see auspice.broadinstitute.org. (**B**) Proportion of genomes that were inferred as imported (ancestral state as not from MA) in the early (prior to March 28, 2020), middle (March 28 - April 14, 2020) and late (after April 15, 2020) time periods of the MA epidemic. (**C**) The proportion of importation events and cases that were associated with singleton introductions (importation events associated with a single case in MA) into the Boston area over sub-sampled trees. (**D**) Allele frequency of the C2416T mutation by state. (**E**) Allele frequency of the C2416T and C26233T alleles in 159,043 GISAID samples reported through October 17, 2020. The vertical black line denotes the end of the business conference on February 27th. (**F**) Time tree of all sequences containing the C2416T variant collected before September 30th 2020

We constructed a phylogenetic tree from this SARS-CoV-2 dataset alone, and additional trees from these data combined with repeated subsampling (Fig. 2A) from the Global Initiative on Sharing All Influenza Data (GISAID) (Materials and Methods). These trees form the basis of our analysis of the Boston area epidemic. The presence of a temporal signal in our dataset (fig. S6) means that a molecular clock can be fitted to infer the timing of ancestral branching based on the SARS-CoV-2 genomes.

We identified putative introductions into the Boston area by carrying out ancestral state reconstruction for these phylogenetic trees (Materials and Methods). In total, we identified more than 122 [95% CI 122 - 161, median 143] putative introductions into the Boston area through May 9, stemming from sources on four continents (Table 1 and fig. S7, A and B). We characterize these introductions as putative because detailed ancestral reconstruction is limited by gaps in the global record of available genomes (*12*), and because the time scale of migration (hours to days) may exceed the rate of viral evolution (~1 new substitution every 13 days). Most of these inferred introductions occurred early in the pandemic, in March and early April, primarily from elsewhere in North America and from Europe (Table 1 and Fig. 2B). We observed close phylogenetic relatedness between genomes from the Boston area and genome sequences from elsewhere in the northeastern and eastern USA (fig. S8), consistent with frequent domestic travel that continued even after international routes were largely closed. The fraction of cases that were imported decreased over time (Fig. 2B), with the steepest decline during March (Fig. S9), likely reflecting the expansion of existing local clades as the outbreak accelerated and travel restrictions were implemented. By April 2020, the vast majority of cases (median 90.7%, 89.2 - 91.9%, 95% CI) resulted from local populations, rather than from new importations (Table 1, Fig. 2B, and fig. S9).

**Table 1:** Estimate of SARS-CoV-2 introductions into Massachusetts. Results of ancestral trait inference using a binary model (MA vs non-MA) and regional model (regional geographic categories) are shown. 95% confidence intervals are shown in parentheses and derived from subsampling the database of global strains (Materials and methods).

Region	Before 28 March	28 March to 15 April	After 15 April
Binary model			
Non-MA	76 (61 to 86)	40 (33 to 46)	28 (23 to 33)
Regional Model			
Africa	0 (0 to 1)	0 (0 to 1)	0 (0 to 1)
Asia	2 (1 to 4)	0 (0 to 1)	1 (0 to 2)
Europe	11 (7 to 16)	6 (3 to 9)	2 (0 to 3)
North America	56 (43 to 66)	29 (22 to 34)	22 (17 to 28)
Oceania	0 (0 to 0)	0 (0 to 0)	0 (0 to 1)
South America	1 (1 to 1)	0 (0 to 0)	0 (0 to 0)

**Fig. 3:**
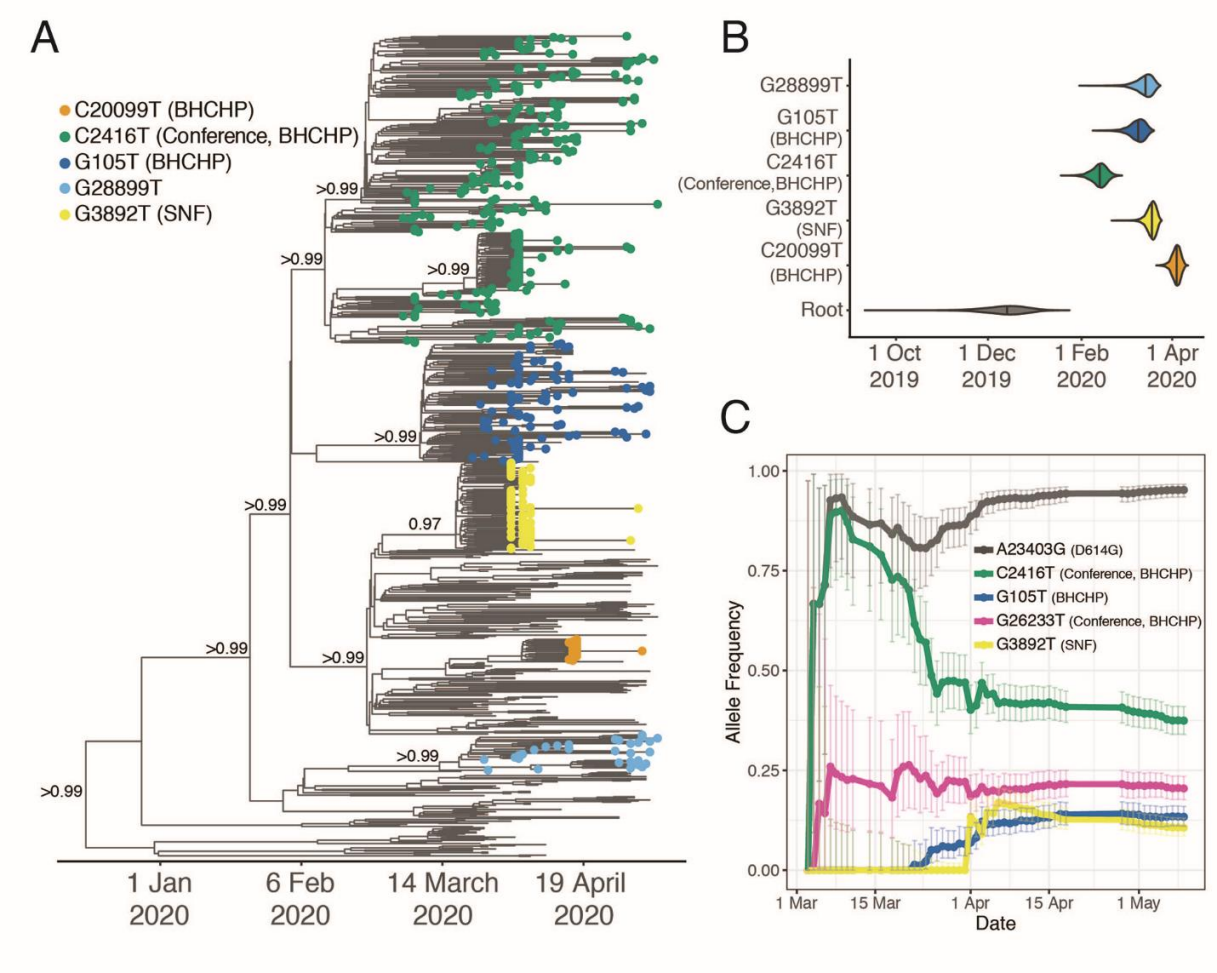
SARS-CoV-2 spread in the Boston area. (**A**) Time-measured maximum clade credibility tree of 772 MA genomes with tips labeled by clade. Nodes with posterior support > 0.8 are labeled. (**B**) Violin plots of tMRCA for the major Boston-area clades. (**C**) Estimated allele frequency in sequenced genomes over time for major Boston-area clades. We use the following abbreviations; Boston Healthcare for the Homeless Program (BHCHP); Skilled Nursing Facility (SNF); large international business conference (Conference).

The majority of cases in our dataset are associated with a minority of importation events: only 29% (26-32%, 95% CI) of importations involved more than one case, but those 29% accounted for 85% (78-88%) of the cases in our dataset (Fig. 2C and fig. S9C). As expected, early importation events resulted in large clades (fig. S9, B and C)—likely due to a combination of longer time to expand and unchecked spread before public health measures were implemented. Several clades established early in the Boston area showed continued community transmission throughout the study period (Table 2 and Fig. 3A), with the lineage containing C2416T, associated with a superspreading event early in the epidemic (described below), being the largest. The C2416T lineage was likely the first of these clades imported into Boston (median estimated time to the most recent common ancestor (tMRCA), February 14, 2020; 95% highest posterior density (HPD) February 4 - 20, 2020) (Fig. 3B). The other four major lineages (G3892T, G105T, G28899T, and C20099T) appeared to enter the region between March and early April 2020. These major lineages, including the superspreading event-associated viruses, circulated widely in the Boston area (fig. S10). This included the communities of Chelsea, Revere, and Everett, which were among the most deeply affected in the state (fig. S11). Consistent with a larger global trend (*13*, *14*), we observed a rise in frequency of viruses harboring the D614G amino acid polymorphism in the Spike protein, conferred by a SNP at nucleotide 23,403 in the Wuhan reference strain, which rose to near-fixation in our dataset by the end of the study period (Fig. 3C) and is present in all of the dominant lineages.

**Table 2:** Major Boston-area lineages identified by lineage-defining mutation.

Lineage	Root	C20099T	G3892T	C2416T	G105T	G28899T
Number of genomes	772	21	77	288	98	34
Epidemiology		BHCHP	SNF	Conference, BHCHP	BHCHP	
Amino acid substitution		ORF1b: A2211V; NSP15: A160V	ORF1a: E1209D; NSP3: E391D			N: R56I, ORF14: E56*
Median tMRCA (95% HPD)	15 December 2019 (20 November 2019 to 4 January 2020)	4 April 2020 (30 March 2020 to 8 April 2020)	19 March 2020 (13 March 2020 to 23 March 2020)	14 February 2020 (4 February 2020 to 20 February 2020)	10 March 2020 (1 March 2020 to 16 March 2020)	15 March 2020 (4 March 2020 to 21 March 2020)

Based on tMRCA estimates for the major Boston-area clades, we do not find evidence of cryptic transmission in the region before mid-February, and none of the importation events we inferred (Table 1) occurred prior to known cases. However, Since testing for SARS-CoV-2 in MA was restricted to a narrow definition prior to established community spread (*15*), we cannot rule out the possibility that isolated importation events and small outbreaks may have escaped detection with the current resolution of sampling.

## Spread of SARS-CoV-2 at an international business conference

Sustained local transmission of SARS-CoV-2 in the Boston area was first detected in early March, and with it case clusters began to appear. The first large cluster was recognized in the context of an international business conference held in Boston from February 26 - 27 (*8*). Public health investigation with contact tracing identified approximately 100 cases associated with this conference (*16*), raising suspicion that a superspreading event had occurred there. We sequenced SARS-CoV-2 genomes from 28 of these cases. These genomes indeed showed the signature of superspreading: they form a tight phylogenetic cluster of highly similar viruses within a narrow time window.

All 28 conference-associated genomes were collected between March 5th and 11th and form a well-supported monophyletic cluster (posterior probability > 0.99) (Fig. 3A and fig. S12) marked by the presence of the SNP C2416T (Fig. 3A). The parent lineage of C2416T, defined by G25563T, was widely distributed in Europe in January and February 2020. The C2416T variant can serve as a marker for tracking the spread of SARS-CoV-2 from the conference, within MA and the USA: it is first reported in the USA in patients associated with the conference and there is no evidence that it had entered the country independent of its appearance there. In our dataset, all C2416T-containing viruses collected prior to March 10^th^ were sampled from individuals with conference exposure, and it was not seen in other publicly available genome data from cases anywhere in the US prior to March 7th, when it appeared in cases that were also likely associated with the conference (*17*). Prior to that, it is seen in the global GISAID database only in 2 French patients, ages 87 and 88, on February 29, 2020 (Fig. 2E). The estimated tMRCA for C2416T-containing genomes is February 14 (95% HPD February 4 - February 20). Taken together, this strongly suggests low-level community transmission of C2416T in Europe in February 2020 before the allele came to Boston via a single introduction, which was then amplified by superspreading at the conference.

We also identified a second variant, G26233T, with a strong conference association. Evidence suggests G26233T emerged during (or theoretically, immediately after) the conference, as it was first seen in 7 of 28 individuals with known conference exposure, including in one sample at intermediate frequency (26%). It is not seen elsewhere in any public genome databases prior to cases associated with the conference (Figs. 2E and 3C). The presence of these two genetic signatures–C2416T in all conference-associated genomes in our dataset, and G26233T in a subset of them–with little or no evidence of transmission prior to the conference, provide markers to track the onward spread of SARS-CoV-2 from the event (Fig. 2F).

The conference-associated lineage was the most common one in our dataset, with C2416T representing 35% (261/744) and C2416T/G26233T representing 20% (151/744) of genomes (excluding those known to be directly associated with the conference). SARS-CoV-2 containing the C2416T allele spread extensively in the Boston area (Fig. 3C and fig. S10A), accounting for between 30% and 46% of genomes from the four counties that make up the Boston area; by the end of the study period, these four counties had reported 51,718 cases. The allele was already at high frequency, in fact, by the time it became clear that an epidemic was underway in the region (fig. S13B), establishing the conditions for extensive spread within Massachusetts and elsewhere.

C2416T began to appear in multiple other US states in early March and increased rapidly in frequency (Fig. 2D and figs. S14 and S15). The effect of this spread was long-lasting. By November 1 2020, viruses containing C2416T could be found in 29 states (fig. S15), and this lineage contributed 1.9% (675/35,566) of all US SARS-CoV-2 genomes in GISAID. States with the largest numbers of cases included ones with known travel by or reported epidemiological links to conference participants returning from the meeting, including Florida, (125/1552 genomes contain C2416T), North Carolina (*18*) (20/94 genomes), and Indiana (*19*) (10/42 genomes) (fig. S15A).

Two additional lines of evidence suggest that the conference superspreading event in Boston contributed substantially to the spread of C2416T outside Massachusetts. First, the C2416T/G26233T sublineage, which arose in the context of the conference, was exported from Boston to at least 18 US states as well as to other countries, including Australia, Sweden, and Slovakia (Fig. 2, D and F, and fig. S14A), with evidence of community spread in many places (fig. S15, C, D, and K). Second, there is evidence from other non-conference associated C2416T sublineages that additional importations from Europe were not major contributors to C2416T prevalence in the US. Two sub-lineages (C2416T/G8371T and C2416T/G20578T) appear frequently among European SARS-CoV-2 genomes in GISAID (295 genomes and 312 genomes, respectively), but are extremely rare among genomes from the US (4 and 1 genomes, respectively) (fig. S14, B and C). This evidence, along with the epidemiological data connecting multiple conference-linked cases to other US states (*18*–*21*), suggests that most C2416T viruses in the US likely derive from this initial introduction.

Genome data reveal that the impact of the conference was far larger than the approximately 100 cases directly associated with the event. Using state-reported case counts, we estimate that by the end of the study period, approximately 50,000 diagnosed cases [44,000 - 56,000] in the US resulted from conference-associated viruses; of these, 46% [40.4 - 51.8%] were in Massachusetts. Through November 1, 2020, we estimate that a total of 245,000 [205,000 - 300,000] cases marked by C2416T, and 88,000 [56,000 - 139,000] cases marked by G26233T, were linked to the conference in the United States. While Massachusetts accounted for most early spread related to the conference, Florida accounted for the greatest proportion of cases overall (29.2% [22.8 - 36.0%], S15G).

While we have attempted to adjust for geography (by using state-level data) and time period as potential confounders, we note that the accuracy of these estimates is limited by the available data: 1) GISAID is not a random sample of the US epidemic, leading to unknown biases in the estimates; 2) existing state-level data are too sparse for detailed spatiotemporal modeling; 3) we have omitted states with ten or fewer available genomes, leading to possible underestimation; 4) diagnosed cases substantially underestimate true incidence (*22*), and 5) the estimates do not account for subsequent transmission of the virus (e.g., 4 million new infections in the US in November 2020). While these estimates are provisional, they convey the likely scope of regional, national, and international spread resulting from a single superspreading event early in the pandemic.

## Spread of SARS-CoV-2 In a skilled nursing facility

**Fig. 4:**
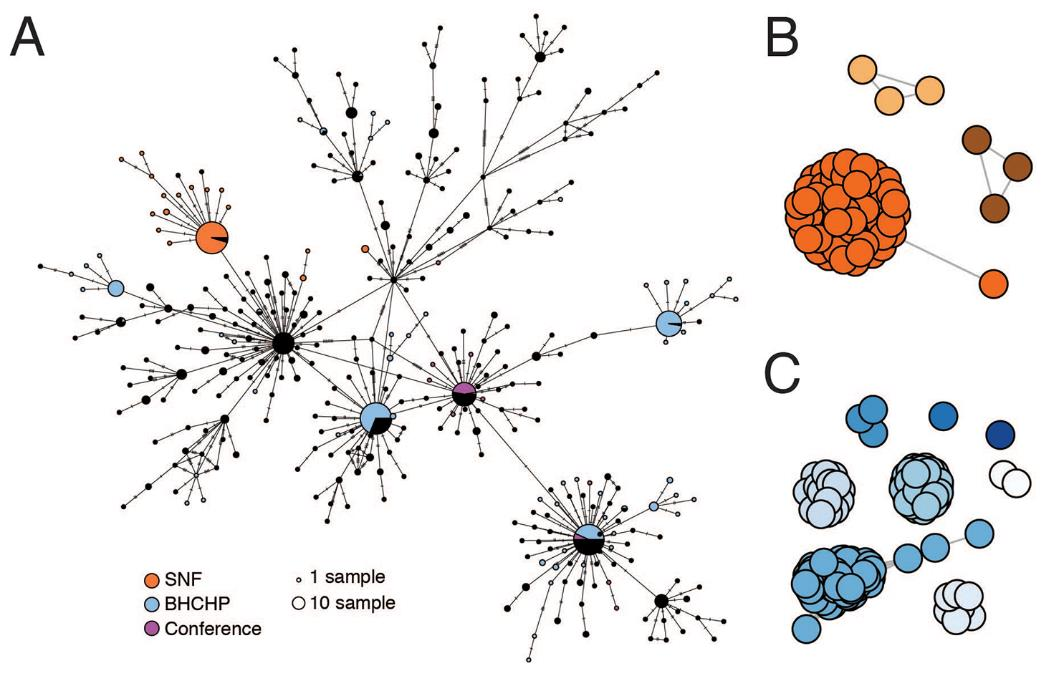
SARS-CoV-2 superspreading events. (**A**) Minimal spanning network showing genetic similarity of SARS-CoV-2 genomes in the MA dataset, with genomes from major known superspreading events highlighted. (**B** and **C**) Gene graphs showing clusters of highly similar sequences among viral genomes from the SNF (B) and BHCHP (C) cohorts. Sequences are clustered when they are separated by < 4 SNPs, and the lengths of lines between points reflect genetic distance.

We investigated a second large cluster of cases, this time at a skilled nursing facility (SNF) in the Boston area, that also proved to involve a superspreading event. The cluster was discovered accidentally: screening of residents prior to a planned relocation in early April revealed widespread infection, and ultimately 85% (82/97) of the residents and 37% (36/97) of the staff (*23*) tested positive for SARS-CoV-2, even though none were known to be symptomatic when screening began. From these individuals we assembled 83 SARS-CoV-2 genomes, 75 of which were found to comprise a single cluster, part of the G3892T lineage described above (Fig. 3A). There was very little genetic variation within the cluster and 59 of the genomes were identical (Fig. 4A), suggestive of a superspreading event. The estimated tMRCA for the cluster of March 20 (Fig. 3B, 95% HPD: March 13 - March 24, 2020), along with the high proportion (30/45) of residents who tested negative on April 1, 2020 but were found to be positive 5 days later (*23*), suggests rapid spread within the facility in late March and early April 2020. Like other outbreaks reported from nursing facilities, the mortality rate was high. While spread outside the facility appeared rare, as only 1% (2/194) of samples in our dataset after April 15, 2020 harbored G3892T, twenty-four residents who tested positive for SARS-CoV-2 died within two weeks of testing.

In addition to the major cluster, another 1-2 small clusters can be seen among the patients and staff in the SNF (95% HPD 2-3 total importations, Fig. 4, A and B, and fig. S16). The different outcome of the introductions—one leading to massive spread within the facility (90% of sampled genomes) and the other(s) to little spread (10% of sampled genomes)—illustrates how superspreading can dramatically impact the transmission dynamics of SARS-CoV-2, and how under the right circumstances it can amplify the effect of any given introduction and associated lineage. Notably, these introductions occurred despite infection control policies—including a restriction on visitors (*24*), universal masking for all staff, masking for all residents when leaving their rooms, and vigilance with hand hygiene—in place for at least two weeks before the first detected infection (*23*).

Upon examination, we concluded that the genetic diversity in the main SNF cluster was strikingly low even under the assumption of recent transmission from a single source. The 18 mutations seen in the cluster are significantly fewer than expected based on the conference cluster (*P* = 0.019), which occurred over a similarly short time window, and much lower than the ~32 mutations expected under a simple model of SARS-CoV-2 substitution (*P* = 0.009, Materials and Methods). This discrepancy might have resulted from low diversity in the SNF index patient, but it may also hint that heterogeneous mechanics of superspreading were at work in the two events. For example, if more virions than usual were transmitted from the SNF index patient to each secondary case — such as through unusually close or prolonged contact, or the initial case having a very high viral load at the time — then we would expect that the resulting infections would more often have the same consensus genome as the index case.

## Cluster investigations in other close contact settings

We studied several additional case clusters with the goal of providing viral genomic data to support public health investigations. These included potential transmission in homeless shelters and within a hospital. First, we analyzed the introduction and spread of SARS-CoV-2 among guests and staff at homeless shelters affiliated with the Boston Health Care for the Homeless Program (BHCHP). We produced 193 complete genomes from 314 samples collected in March and April 2020, including those collected during universal screening at Boston’s largest homeless shelter (*5*). Based on the position of these 193 SARS-CoV-2 genomes from BHCHP in the overall Boston-area tree (Fig. 3A), we identified at least 14 introductions into the BHCHP community (95% HPD 14-18). Of these, 4 resulted in clusters consistent with superspreading, each containing 20 or more highly similar viral genomes (Fig. 4, A and C, and fig. S16B). Two of the clusters descended from the conference-associated C2416T lineage, including one that contained C2416T/G26233T. In total, 54% (105/193) of the genomes in this cohort contained C2416T, of which half (54/105) also contained G26233T, demonstrating that BHCHP guests and staff were affected by community transmission resulting from amplification and spread of conference-associated SARS-CoV-2.

The other two case clusters occurred at Massachusetts General Hospital, where the Infection Control Unit sought genomic data to inform their investigations of possible nosocomial outbreaks. In the first cluster, two patients in the same hospital ward tested positive for SARS-CoV-2 during their hospital stay, after testing negative at the time of admission. In the second, unrelated cluster, four patients who received care in a specialty ward were diagnosed with SARS-CoV-2 infections over a period of several days. For each cluster, complete genomes (2 of 2 from the first cluster and 4 of 4 from the second cluster) were genetically very distinct, a pattern inconsistent with having been infected from the same source during hospitalization (fig. S17). Although we cannot exclude the possibility of nosocomial transmission per se because independent introductions from multiple asymptomatic staff could theoretically have occurred, this demonstrated that the individuals in each cluster were not part of the same transmission chain.

## Conclusions

Genomic analysis of the first wave of the COVID-19 outbreak in the Boston area provides powerful evidence of the importance of superspreading events in shaping the course of this pandemic. In this study we show that importation events occurred very frequently—we identified over 120 independent introductions during the three-month study period—and that they varied enormously in terms of their subsequent impact on local transmission. Consistent with an over-dispersed offspring distribution for SARS-CoV-2 (*25*), in our dataset, a small minority of importations accounted for the majority of observed cases. At least some of this variation in clade sizes results from superspreading events amplifying some lineages and not others. This can be seen in microcosm in one of the two superspreading events we studied in detail: SARS-CoV-2 was introduced at least twice into the skilled nursing facility; one introduction led to widespread transmission and numerous deaths, while the other 1-2 introductions led to a total of six cases.

The other superspreading event, which occurred at an international business conference early in the local epidemic, had a much greater impact on community transmission. Because SARS-CoV-2 viruses circulating at the conference happened to be marked by distinct genomic signatures, we were able to track its downstream effects far beyond the superspreading event itself, tracing the descendants of the virus as they made a large contribution to the local outbreak in the Boston area and as they spread throughout the US and the world, likely causing hundreds of thousands of cases. The different genetic diversity seen in the two events raises the possibility that superspreading encompasses varied transmission dynamics.

Not all case clusters were the result of superspreading. Both hospital clusters consisted of unrelated cases that happened to occur in close proximity to one another. Cases associated with the homeless shelters likely resulted from a mix of superspreading events and more general transmission, although we lack the detailed epidemiological data to explore their history in depth. Where we were able to study superspreading events in detail, in the SNF and the conference, it was not because they were unique in size or character, but because circumstances allowed close study. For both, we had dense sampling during a narrow time window of a clearly demarcated exposed population, aided by good data on prevailing genetic diversity to provide context.

Our findings highlight the close relationships between seemingly disconnected groups and populations: viruses from international business travel seeded major outbreaks among individuals experiencing homelessness, spread throughout the Boston area including to other higher risk communities, and were exported to other domestic and international sites. It also illustrates the role of chance in the trajectory of an epidemic: a single introduction had an outsize effect on subsequent transmission because it was amplified by superspreading in a highly mobile population very early in the outbreak, before many public health precautions were put in place, and when its effects would be further amplified by exponential growth and subsequent superspreading events (e.g., among the homeless). By contrast, other early introductions led to very little onward transmission, and the superspreading event in the SNF, while devastating to the residents, had little large-scale effect because it occurred later and in a more isolated population. While superspreading events among medically vulnerable populations, such as nursing home residents, have a larger immediate impact on mortality, our findings raise the possibility that—paradoxically—the implications may be greater, when measured as a cost to society, for superspreading events that involve younger, healthier and more mobile populations because of the increased risk of subsequent transmission. With the possibility of vaccines that protect against disease but not infection, this consideration may be increasingly important. In summary, this study provides clear evidence that superspreading events may profoundly alter the course of an epidemic and implies that prevention, detection, and mitigation of such events should be a priority for public health efforts.

## Materials and Methods

### Sample and data collection

This study was approved by the Partners Institutional Review Board under protocol 2019P003305 and MDPH IRB 00000701. We obtained samples and selected metadata from the MGH Microbiology Laboratory and MADPH under a waiver of consent for viral genome sequencing. All samples were nasopharyngeal (NP) swabs that tested positive for SARS-CoV-2 by RT-qPCR. Epidemiological data on exposure and geography were obtained from medical record review (MGH) or collected by the DPH laboratory in the process of clinical testing. Samples included individuals with known exposures to suspected superspreading events and individuals where no possible exposures were known. We compared known information about these cases to publicly available daily and weekly data on cases of SARS-CoV-2 in MA for the period January 1 - August 1 (https://www.mass.gov/info-details/covid-19-response-reporting).

### Viral sequencing and analysis

Total RNA was extracted from inactivated NP swabs and presence of virus was confirmed using an RT-qPCR assay detecting the N1 gene of the virus. Metagenomic sequencing libraries were prepared as previously described (*26*)). Briefly, following DNase treatment to remove residual DNA and depletion of human rRNA, cDNA was synthesized using random hexamer priming. Illumina sequencing libraries were prepared from cDNA and sequenced with 100-nucleotide paired-end reads.

We conducted all analyses using viral-ngs 2.0.21 on the Terra platform (app.terra.bio). All of the workflows named below are publicly available via the Dockstore Tool Registry Service (dockstore.org/organizations/BroadInstitute/collections/pgs). Code is also archived at doi:10.5281/zenodo.4306358 and doi:10.5281/zenodo.4306362. Briefly, samples were demultiplexed (demux_only workflow), filtered for known sequencing contaminants and SARS-CoV-2 genomes were assembled using a reference-based assembly approach (assemble_refbased) with the reference genome NC_045512.2. Following a stringent quality control and filtering, we identified a final set of 772 high-quality assemblies from unique individuals that was used for all subsequent analyses and deposited in GenBank and GISAID. We used R (*27*), Bioconductor (*28*), ggplot2, tidyverse (*29*), and ggtree (*30*) to clean and plot data and trees, and choroplethr to draw maps.

To detect the presence of 20 common respiratory viruses in sequenced samples, we used Kraken2 (*31*) implemented in the *classify_single* and *merge_metagenomics* workflows. A virus was determined to be present if more than 10 reads mapped to that species. Wherever possible, these co-infections were confirmed using the BioFire FilmAssay Respiratory Panel.

We constructed phylogenetic maximum likelihood (ML) and time trees with associated visualizations using the Augur pipeline (*augur_with_assemblies*) and SARS-CoV-2-specific procedures taken from github.com/nextstrain/ncov for our 772 genomes and a representative background set of 4,011 subsampled from the GISAID database on 15 June, 2020. We separately constructed ML trees from trimmed alignments to estimate root-to-tip distances and obtain branch support for ML phylogenies. To estimate coalescence dates of major lineages we constructed Bayesian time-trees using BEAST 2.6.2 with a general time reversible substitution model with 4 rate categories drawn from a gamma distribution (GTR4G), a strict clock, coalescent exponential tree prior, a uniform [-inf, inf] prior for the clock rate, a 1/x [-inf, inf] prior for the coalescent exponential population size; and a laplace [-inf, inf] prior for the growth rate.

### Ancestral state reconstruction

We used 3 orthogonal approaches to reconstruct the ancestral location of unsampled nodes: 1) a ML approach using the augur pipeline, 2) a maximum parsimony approach using the Narushima and Hanazawa method as implemented in the MPR function of the ape package in R, and 3) a bayesian approach using BEAST1.10.4. In each case, we use a binary classification of “MA” vs “non-MA” to identify nodes that represent a likely importation event into Massachusetts. For full details of each approach see the Supplementary Materials and Methods.

### Analysis of superspreading events

To estimate the number of cases linked to the conference we estimated the proportion of genomes with C2416T and C2416T/G26233T per state by multiplying the observed proportion in genomes reported in GISAID through November 2nd 2020 by case counts reported in the New York Times COVID data repository (https://github.com/nytimes/covid-19-data). We summed across states using a Monte Carlo simulation (*n* = 10,000).

To show clustering within the SNF and BHCHP cases, we constructed a minimal spanning haplotype network from the trimmed ML alignment of 772 genomes using PopART v1.7 (*32*) with masking of regions where any sequence had ambiguous bases. Gene graphs were constructed using pairwise distance matrices computed on aligned SARS-CoV-2 genomes and clustered using the R package adegenet (*33*). Importations into the SNF and BHCHP populations were calculated using a bayesian approach similar to that described above (see Supplementary Materials and Methods for more details).

We define a superspreading event as the transmission from a single source to a large number of secondary infections, where the number is large enough that it would occur < 1% of the time in a simple Poisson model of transmission (*34*). For this study, using an R_eff_ value of 3.0, we set the threshold at a minimum of nine transmissions. We compared the number of mutations among conference-associated and SNF-associated genomes with the expected number based on a generation time of 5.0 days (*35*) and a mean substitution rate of 1.04 × 10^−3^/bp/year (fig. S6C) and calculated a p-value based on the fraction of draws yielding fewer mutations than observed.

## Supplementary Materials

science.sciencemag.org/cgi/content/full/science.abe3261/DC1 Materials and Methods Figs. S1 to S17 References (*36*–*43*) Tables S1 to S3 MDAR Reproducibility Checklist
